# Molecular Simulation of the Effects of Cyclic Organic Compounds on the Stability of Lccbm Hydrates

**DOI:** 10.3390/molecules27207077

**Published:** 2022-10-20

**Authors:** Wenbo Lv, Cunbao Deng, Zhixin Jin, Hao Zhang, Yansheng Wang

**Affiliations:** 1 College of Safety and Emergency Management and Engineering, Taiyuan University of Technology, Jinzhong 030600, China; 2 Datong Coal Mine Group Co., Ltd., Datong 037037, China

**Keywords:** gas hydrate, low-concentration coal bed methane, gas separation, cyclic organic compounds, molecular dynamics, stability

## Abstract

CH_4_ can be separated from low-concentration coal bed methane (LCCBM) by using the hydrate-based gas separation (HBGS) method. To study the contribution of different cyclic organic compounds to the separation of CH_4_ in LCCBM, an LCCBM hydrate model was constructed. Based on the Monte Carlo and molecular dynamics theory, we simulated the effect of three cyclic organic compounds—cyclopentane (CP), cyclopentanone (CP-one), and cyclopentanol (CP-ol)—on the stability of the LCCBM hydrate at P = 2 MPa, various temperatures, and discussed the structural stability of the hydrate in depth in terms of final snapshots, radial distribution function, mean square displacement, diffusion coefficient, and potential energy change. The results showed that for the CH_4_-N_2_ LCCMM gas mixture, CP showed the best facilitation effect compared to the other two cyclic compounds by maintaining the stability of the LCCBM hydrate well at T = 293 K. The promotion effect of CP-one is between CP and CP-ol, and when the temperature increases to T = 293 K, the oxygen atoms in the water molecule can maintain the essential stability of the hydrate structure, although the orderliness decreases significantly. Moreover, the structure of the hydrate model containing CP-ol is destroyed at T = 293 K, and the eventual escape of CH_4_ and N_2_ molecules in solution occurs as bubbles. The research results are important for further exploration of the mechanism of action of cyclic promoter molecules with LCCBM hydrate molecules and promoter preferences.

## 1. Introduction

Coal mine methane (CMM), also known as coal bed methane (CBM), is an unconventional natural gas resource with global reserves of 2.6 × 10^14^ m^3^, equivalent to 3.15 × 10^14^ kg of standard coal. China’s shallow coal bed methane reserves (burial depth less than 2000 m) are about 3.0 × 10^13^ m^3^, ranking the third largest in the world [1,2]. However, the methane concentration in underground coal mines is usually low due to the inevitable mixing of air during the coal mining process. Most of the low-concentration coal bed methane (LCCMM, 1% ≤ cCH_4_ ≤ 30%) is not utilized and is emitted directly into the atmosphere; such treatment is not only a waste of resources but also has a serious environmental impact due to the greenhouse effect of CH_4_ [3,4,5]. Therefore, the development of efficient LCCBM utilization technologies is important for optimizing the energy mix and reducing greenhouse gas emissions.

Recently, significant progress has been made in hydrate-based gas separation (HBGS) technology to capture CO_2_ from flue gas or fuel gas [6,7], or separate noble gases (Ar, Kr, Xe) from the air [8]. At present, the method has been successfully applied to CH_4_ recovery from LCCBM. The basis of the HBGS method is that under a certain condition, CH_4_ molecules will be preferentially trapped in the empty hydrate cavities while other gas molecules remain free in the gas phase, so CH_4_ can be recovered from LCCBM through the decomposition of gas hydrates [2]. Compared with traditional separation methods such as solution absorption [9], cryogenic liquefaction [10], and membrane-based separation [11], the HBGS method has the advantages of high gas storage capacity, moderate temperature and pressure conditions, inexpensive and environmentally friendly materials, recyclable liquid solution, and superior gas selectivity [12,13,14].

However, forming LCCBM hydrates in pure water requires very high pressures, which significantly increases the economic costs and explosion risks [3]. Therefore, an additive is needed not only to reduce the formation pressure of hydrate and increase its stability but also to capture CH_4_ more efficiently from LCCMM [15,16]. Zhong et al. [17] used a CP (C_5_H_10_) thermodynamic additive to lower the phase equilibrium pressure and increase the operating temperature for hydrate formation to recover CH_4_ from low concentrations of CH_4_/N_2_ coal bed methane. Juan et al. [18] determined the equilibrium conditions of methane hydrate in an aqueous solution with CP-one (C_5_H_8_O) and 4-hydroxy-4-methyl-2-pentanone additives, and the results showed that the CP-one additive promoted the formation of methane hydrate. Li et al. [19] experimentally measured the dissociation points of methane hydrate mixtures in aqueous solutions of acetamide, CP-ol, and 1,3-dioxane and showed that the addition of acetamide inhibited the formation of methane hydrate mixtures, while the addition of CP-ol (C_5_H_10_O) and 1,3-dioxane promoted the formation of methane hydrate mixtures. Molecular dynamics (MD) simulations have been used to provide molecular insights into many important aspects of gas hydrate, including nucleation [20], guest molecule substitution [21], growth and decomposition mechanisms [22], and to assess the role of hydrate promoters [23]. Studies at the molecular level can provide quantitative microscopic insights into kinetic and equilibrium properties, thus facilitating optimization of operating conditions [24]. Gharebeiglou et al. [25] studied 3-membered SII hydrates of methane + cyclic organic compounds (COCs) using molecular dynamics (MD) simulations and showed that the COC guest molecules have a stabilizing effect on the hydrates. Kondori et al. [26] determined the diffusion coefficient, density, and heat capacity of hydrates using the MD method and tested the stability of the water cage under different temperature and pressure conditions. Zhang et al. [27] determined the dynamic and structural properties of methane hydrate by mean square displacement, potential energy, density profile, and radial distribution function (RDF) using the MD method.

In summary, studies such as those by Juan et al. [18], Li et al. [28], and Zhang et al. [27] focused on methane hydrates of single gas components. Although Zhong et al. [17] experimentally investigated multi-component gas hydrates, only a single promoter of CP was selected, lacking comparisons with similar promoters. The cyclic organic compounds have been studied by many scholars as hydrate thermodynamic promoters, but comparisons of the differences in the promoter molecules of similar cyclic structures for LCCBM hydrate promotion and the reasons for these differences have rarely been investigated. In addition, since it is challenging to clarify the interaction between promoter molecules and hydrates by macroscopic experimental methods, it is necessary to establish a model that reflects the actual state of LCCBM hydrates and conduct studies using MD simulations.

In this paper, LCCBM is considered as a mixture of CH_4_ and N_2_ (CH_4_-N_2_, 3:7), and three cyclic compounds, CP, CP-one, and CP-ol were selected as guest molecules to participate in the construction of LCCBM hydrates using the Monte Carlo method. From the perspectives of molecular dynamics and crystal structure at the microscopic level, we compare the different effects of cyclic compounds with similar molecular structures on the hydrate stability of LCCBM and explore their causes to provide theoretical implications for the specific applications and promoter preferences for CH_4_ separation in LCCBM.

## 2. Results and Discussion

### 2.1. Final Snapshots

**Figure 1:**
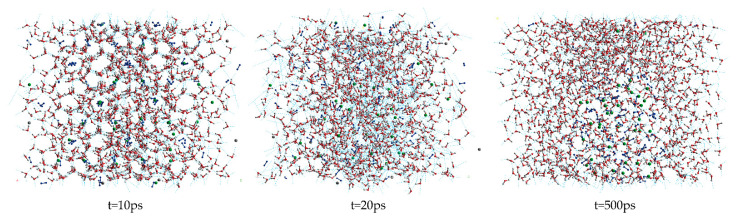
Snapshot structures of molecular dynamic runs to simulate A0 model structure after 500 ps at P = 2 MPa and T = 273 K.

Figure 1 shows the snapshot structures of the A0 LCCBM hydrate model without any promoter in the pure water system, obtained after 500 ps simulation at P = 2 MPa and T = 273 K. At t = 10 ps, the stability of the cage structure of hydrate started to decrease significantly but still maintained the basic structure; however, at t = 20 ps, the hydrogen bonding of hydrate was broken and the cage structure started to collapse. At t = 500 ps, the hydrate cage structure completely disappeared, and the gas molecules started to aggregate into bubbles and disperse in the aqueous solution. This behavior indicates that the LCCBM hydrate model without any promoter is poorly stable and decomposes at lower temperatures.

**Figure 2:**
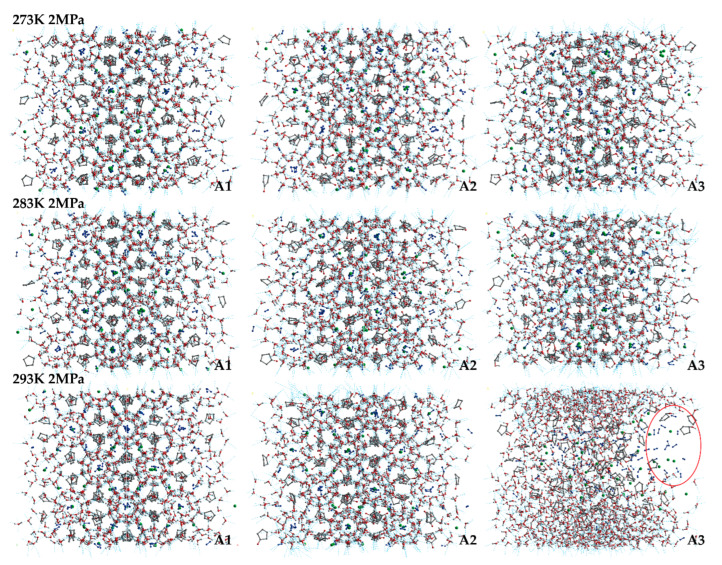
Final snapshots of molecular dynamic runs to simulate **A1**, **A2**, and **A3** model structure after 500 ps at P = 2 MPa and T = 273 K, 283 K, and 293 K. The red circle represents the bubble formed by the accumulation of gas molecules in the system.

The final snapshots of the LCCBM hydrate models containing different promoter molecules after 500 ps for P = 2 MPa, T = 273 K, 283 K, and 293 K are shown in Figure 2. From the snapshots of the structures of the hydrate simulation system in Figure 2, it can be seen that when T = 273 K and the three models A1, A2, and A3 can maintain the original structure with a high order of hydrogen bond overlap, and the guest molecules can coincide concerning the cage cavity center, the oxygen atom coordinates overlap, showing a better network symmetry of the structure itself. The pressure is constant, and the hydrate cage structures of models A1, A2, and A3 all show different degrees of disorder as the temperature increases. At T = 283 K, the cage structure of the LCCBM hydrate containing CP-one and CP-ol of A2 and A3 models shows obvious distortion, and the hydrogen bonding network structure is disordered in a small area, but the gas molecules can still exist in the center of the hydrate cage. However, at T = 293 K, the ordered crystal structure of the A3 model hydrate containing CP-ol no longer exists, and although hydrogen bonds exist, the cage structure of the hydrate has collapsed, and the guest molecules escape and gather to form bubbles (positions marked by red circles in Figure 2). The hydrate has been transformed from a solid to a liquid at this moment, and its original structure has been destroyed.

The overall stability of the LCCBM hydrate model with different cyclic promoters can be visualized from the snapshots of the structures of the simulation, i.e., the model stability A1 > A2 > A3 with increasing temperature at constant pressure, but it is difficult to describe the degree of distortion of the crystal cage quantitatively.

### 2.2. Radial Distribution Function

To further investigate the stability of the LCCBM hydrate cage, we analyzed the microscopic properties of the simulated system. The radial distribution function (RDF) plays a central role in liquid state theories. The *g_αβ_* (*r*) represents the probability of occurrence of atom *β* found at a distance *r* from atom *α*. The radial distribution function is described in Equation (1) as follows:

(1) $$g_{\alpha \beta }(r)=\frac{V}{N_{\alpha }N_{\beta }}\left(\sum_{i=1}^{N_{\alpha }}\frac{n_{i}\beta (r)}{4\pi r^{2}\Delta r}\right)$$

In the formula, V denotes the volume of the simulation box, $N_{\alpha }$ and $N_{\beta }$ refer to the total number of α and β particles, respectively, and $n_{i}\beta (r)$ stands for the total number of apart from the atom at the spherical distance of r. In this study, the goo (oxygen–oxygen) and g_CC_ (carbon–carbon) radial distribution functions were used to simulate the water and methane molecules in LCCBM hydrate systems.

**Figure 3:**
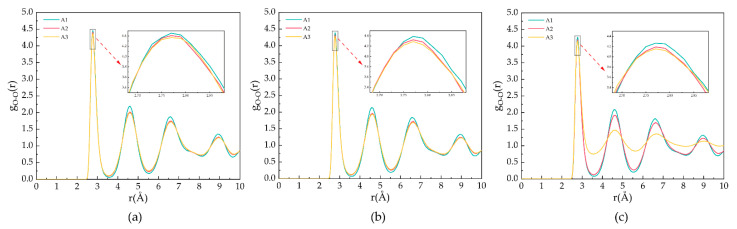
RDFs of oxygen atoms in H_2_O molecules of A1, A2, and A3 model after 500 ps at P = 2 MPa and three different temperatures for (**a**) T = 273 K, (**b**) T = 283 K, and (**c**) T = 293 K.

Figure 3 shows the goo for the A1, A2, and A3 models at a P = 2 MPa and T = 273 K, 283 K, and 293 K. As shown in Figure 3a, T = 273 K, the first maximum peak of oxygen atoms occurs at 2.77 Å, representing the distance between oxygen atoms and the closest water molecules. The second and third peaks appear at 4.57 Å and 6.59 Å, respectively, representing the distance of oxygen atoms in the hydrogen bonding of hydrate cages. The maximum peaks of the A1 model containing CP are larger than those containing CP-one and CP-ol, respectively, and this phenomenon indicates that the highest oxygen atom ordering is found in the A1 model at T = 273 K. As shown in Figure 3b, the peak of goo of the same model becomes lower, but the peak valleys become higher with an increasing temperature at constant pressure. This behavior indicates that the oxygen atoms in the water molecule are less ordered, and the crystal structure of the LCCBM hydrate begins to be gradually disrupted before complete dissociation. With the increasing temperature, the RDF of oxygen atoms in the LCCBM hydrate system containing CP-one is between CP and CP-ol, indicating that the effect of CP-one on the stability of the LCCBM hydrate at P = 2 MPa is between the other two promoters. As shown in Figure 3c, the peak of the A3 model containing CP-ol decreases dramatically at T = 293 K, indicating that the hydrate skeleton is severely disrupted at this time.

**Figure 4:**
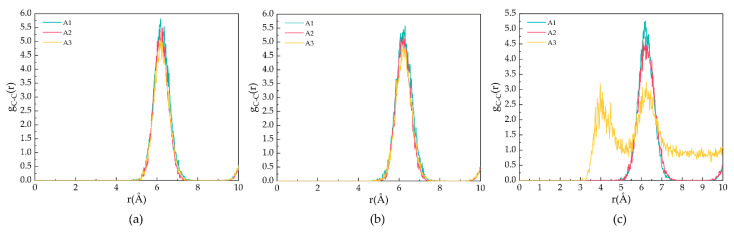
RDFs of carbon atoms in CH_4_ molecules of A1, A2, and A3 model after 500 ps at P = 2 MPa and three different temperatures for (**a**) T = 273 K, (**b**) T = 283 K, and (**c**) T = 293 K.

Figure 4 demonstrates the g_CC_ for the A1, A2, and A3 models at P = 2 MPa and T = 273 K, 283 K, and 293 K. As shown in Figure 4a,b, the peak position is located at 6.21 Å, corresponding to the fact that the CH_4_ molecules are separately trapped in different water cavities of the clathrate lattice. As shown in Figure 4c, at T = 293 K, the first C−C peak of the A3 model contains CP-ol shifts to 4.17 Å, indicating that the CH_4_ molecules escape from the cavity and start accumulating, as demonstrated in the snapshot shown in Figure 1.

### 2.3. Mean Square Displacement

During the simulation, the particles move in the molecular simulation box. Mean square displacement (MSD) is a measure of the average distance a given particle in a system travels. The MSD is represented by the following Equation (2):

(2) $$\mathrm{MSD}=(|{\bar{r}}_{i}-{\bar{r}}_{i0}{|}^{2})=\frac{1}{N}\sum_{i=1}^{N}(|{\bar{R}}_{i}(t)-{\bar{R}}_{i}(t_{0}){|}^{2})$$

In the formula, N represents the total number of particles, ${\bar{R}}_{i}(t)$ represents the position of the particles at time t, and ${\bar{R}}_{i}(t_{0})$ refers to the initial position of the particles. For a stable hydrate crystal, the molecules are at relatively fixed lattice points, and the MSD should fluctuate around a value slightly greater than 0, considering the vibration and rotation of the molecules at the lattice points. When the hydrate decomposes, the positions of its molecules are relatively free, and the MSD increases with the simulation time.

**Figure 5:**
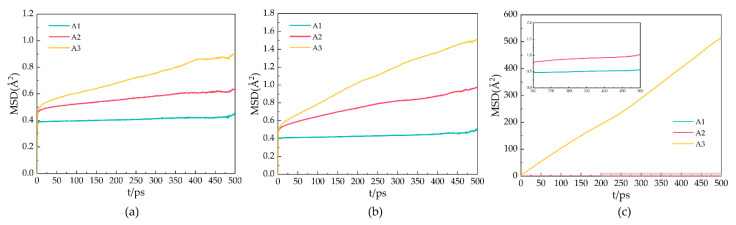
MSDs of oxygen atoms in water molecules of A1, A2, and A3 model after 500 ps at P = 2 MPa and three different temperatures for (**a**) T = 273 K, (**b**) T = 283 K, and (**c**) T = 293 K.

Figure 5 shows the MSDs of oxygen atoms in water molecules after 500 ps for the A1, A2, and A3 models at P = 2 MPa and T = 273 K, 283 K, and 293 K. As shown in Figure 5a, the MSD of the A1 model containing CP is the smallest at T = 273 K. The MSD of the oxygen atoms fluctuates around 0.39 × 10^−18^ m^2^, indicating that the LCCBM hydrate is in the most stable structural state under this condition. As shown in Figure 5b, at T = 283 K, the MSDs of the A2 and A3 models start to move upward, while the A1 model remains almost unchanged, which means that the crystal structures of hydrates containing CP-one and CP-ol gradually become unstable, and the diffusion rate of water molecules becomes higher, while the structures of hydrates containing CP remain stable. As shown in Figure 5c, at T = 293 K, the MSD of A1 changed very little compared to those at T = 273 K and T = 283 K. The MSD of A2 increased to some extent, and the MSD of the A3 model increased dramatically, showing a trend similar to that of the liquid H_2_O molecule.

It can be concluded that the water molecules in the hydrate vibrate and rotate around the lattice. When the hydrate dissociates, the water molecules leave the lattice, and the crystal cage collapses, leading to an increase in the MSDs of the water. This result indicates that the LCCBM hydrate containing CP and CP-one can remain essentially stable at 2 MPa, while the hydrate containing CP-ol is completely structurally destroyed at P = 2 MPa and T = 293 K.

### 2.4. Diffusion Coefficient

The diffusion coefficient D is calculated from the results of the mean square displacement combined with Einstein’s algorithm. The stability of gas hydrates can be studied by calculating the diffusion coefficient of water molecules in the hydrate, which can be expressed by the following Equation (3):

(3) 6Dt=MSD

In the formula, D denotes the diffusion coefficient, t refers to the simulation time, and MSD denotes the mean square displacement. The diffusion coefficient value for solid molecules is close to 0, and the value of the diffusion coefficient for gas molecules tends to infinity.

**Figure 6:**
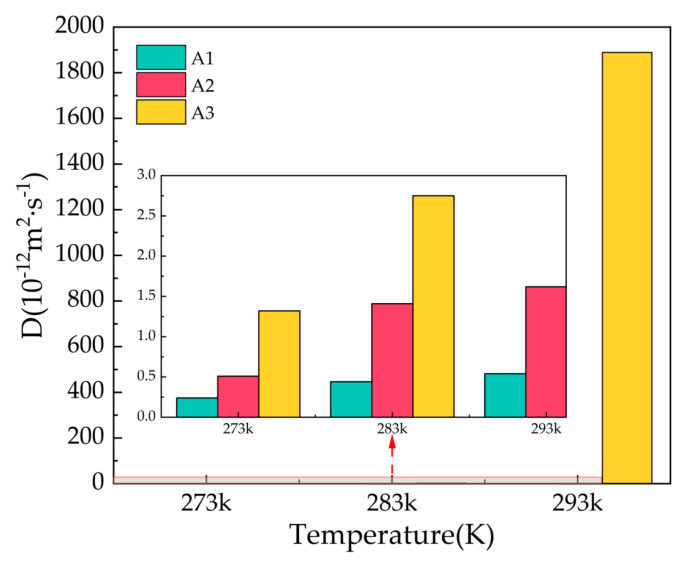
Diffusion coefficients of oxygen atoms in water molecules of A1, A2, and A3 model after 500 ps at P = 2 MPa and T = 273 K, 283 K, and 293 K.

Figure 6 shows the diffusion coefficients of oxygen atoms in water molecules after 500 ps for the A1, A2, and A3 models at P = 2 MPa and T = 273 K, 283 K, and 293 K. At T = 273 K, the A1 model containing CP has the smallest diffusion coefficient, D = 0.24 × 10^−12^ m^2^/s, which is approximately equal to the solid diffusion coefficient. With the increase in temperature, the diffusion coefficients of A1, A2, and A3 models all increase to different degrees, with smaller increases in the A1 model, and the A3 model containing CP-ol at 293 K shows dramatic increases, implying that the water molecules forming hydrogen bonds are active, the hydrogen bond network is not fixed, and the bond energy is reduced.

Combining the information from the final snapshots, RDFs, and MSDs, it can be seen that CP is most effective in maintaining the cage structure of the hydrate at P = 2 MPa, while the structure of the LCCBM hydrate containing CP-ol is in an unstable state at T = 293 K, and the length of the hydrogen bonds is uncertain and changes from solid to liquid with simulation time.

### 2.5. Potential Energy

**Figure 7:**
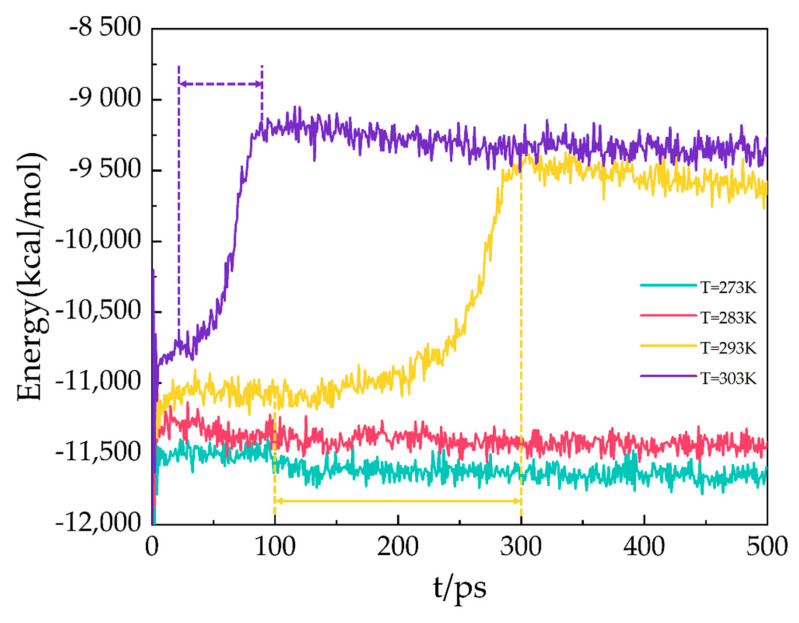
Potential energy for A3 models at P = 2 MPa and T = 273 K, 283 K, 293 K, and 303 K.

In the MD simulation, the potential energy consists of long-range Coulombic and van der Waals interactions. As shown in Figure 7, the potential energy of the A3 LCCBM hydrate model at P = 2 MPa and T = 273 K, 283 K, 293 K, and 303 K varies as a function of simulation time. At the beginning of the simulation, the hydrogen bonds between the water molecules constituting the hydrate cage structure are not disturbed before decomposition because the molecules of H_2_O, N_2_, CH_4_, etc., vibrate and rotate, and the potential energy changes around the equilibrium value. At T = 273 K and 283 K, the potential energy of A3 models constantly fluctuates around a value, and the energy does not increase significantly and remains stable. At T = 293 K, the A3 model containing CP-ol has a dramatic increase in potential energy within 100–300 ps, which means that the hydrogen bonds between water molecules in the hydrate cage are broken down in the range of 100–300 ps. This leads to the starting moment of dissociation of the A3 model at about 100 ps. The structure of the LCCBM hydrate is completely decomposed after 200 ps, and the potential energy stops changing and oscillates around a value. Furthermore, at T = 303 K, the potential energy increases dramatically in the range of t = 25–95 ps, which means that the model is completely decomposed within 70 ps.

The results show that the LCCBM hydrate containing CP-ol decomposes at P = MPa and T = 293 K. Moreover, as the temperature increases, the hydrate starts to decompose earlier, and the time required for complete decomposition is shorter.

**Figure 8:**
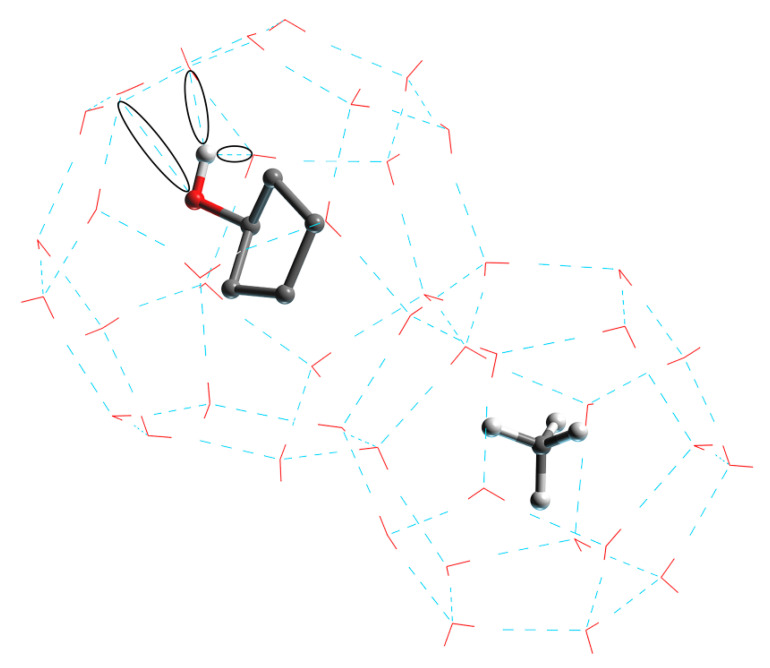
Structure of LCCBM hydrate cages in the presence of CP-ol molecules with new hydrogen bonds between water and CP-ol molecules.

Figure 8 shows the new hydrogen bonding in the form of black circles. The hydrophilic hydroxyl group in the CP-ol molecule in the large crystal cage combines with the water molecule inside the crystal cage to form a new hydrogen bond. The hydrogen bonding between the functional group and the water molecule becomes stronger when the polarity of the functional group becomes higher, and the currently known order of polarity of the functional group is alcohol > ketone > alkane [29,30]. These new hydrogen bonds break and weaken the primary (or primary) hydrogen bonds between water molecules in the hydrate structure, and the large crystal cages in turn have a greater effect on the hydrate structure than the small ones, leading to a significant decrease in structural stability. Thus, the most polar of the three cyclic compounds, CP-ol, shows the worst hydrate stability, followed by CP-one and CP.

## 3. Simulation Details

### 3.1. Model Construction

Previous studies have shown that large promoter molecules such as CP can form SII hydrates with small gas molecules such as CH_4_, N_2_, and CO_2_, and large molecules occupy 5^12^6^4^ large cage cavities and small molecules occupy 5^12^ small cage cavities [31,32,33]. In this paper, these models were constructed using Material Studio 8.0. The unit cell of SII hydrate was constructed based on the positions of the oxygen atom in the water molecule, which are determined by X-ray diffraction experiments [34]. The hydrogen atoms were then randomly added and reoriented to satisfy Bernal–Fowler’s “ice rule” [35]. A 2 × 2 × 2 supercell was built by replication of a unit cell to create a simulation box with 34.35 Å × 34.35 Å × 34.35 Å size, and the angle of the model was α = β = γ = 90° in the space group Fd3m, in which 1088 water molecules form the main framework of 128 small cage cavities and 64 large cage cavities. Periodic boundary conditions were applied to all three dimensions of the system.

**Figure 9:**
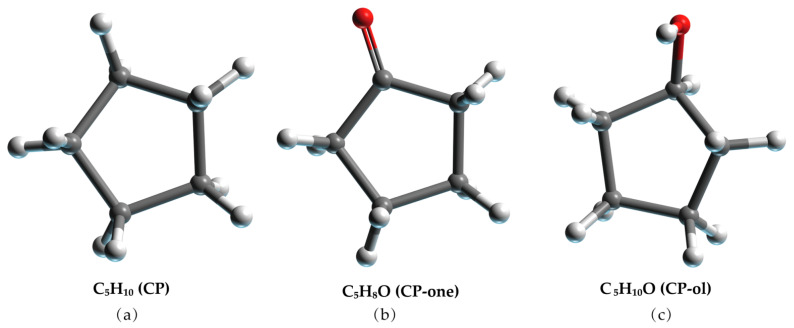
Molecular model of (**a**) CP, (**b**) CP-one, and (**c**) CP-ol. The gray, red, and white balls represent C, O, and H atoms, respectively.

**Figure 10:**
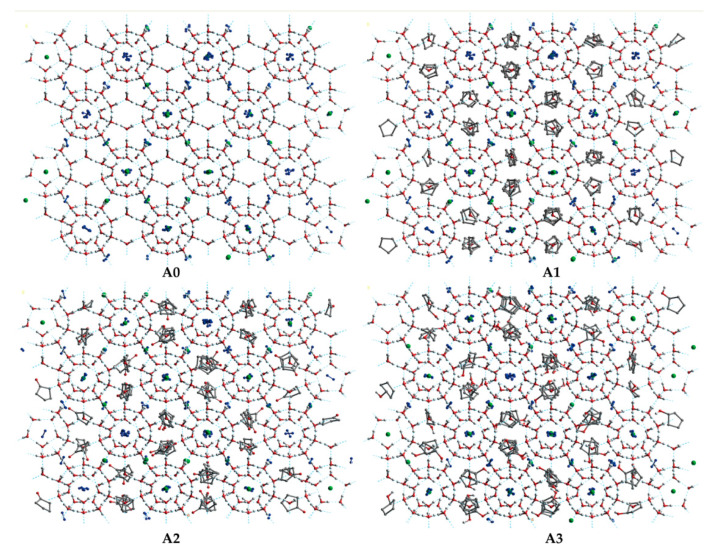
The initial structure of the (**A0**), (**A1**), (**A2**), and (**A3**) model. The gray, red, and white balls represent C, O, and H atoms, respectively. The green balls represent CH_4_ molecules.

The SII LCCBM hydrate models containing three different cyclic promoters were constructed using the Monte Carlo [36] method of the adsorption module for the adsorption of guest molecules. In this case, the Metropolis method [37] was applied, considering only the position and conformation of the adsorbate and treating it as a rigid sphere. Moreover, the grand canonical ensemble (VTμ) was used in the Monte Carlo simulations to represent a determined volume (V), temperature (T), and chemical potential (μ). CH_4_ and N_2_ molecules were randomly placed inside all 128 small cage cavities, while the large cage cavities were filled with 64 CP, CP-one, and CP-ol molecules, respectively (see Figure 9). In addition, we also constructed a model of the LCCBM hydrate in a pure water system without any promoter as a control group. The above models fully considered the stochastic nature of the actual formation process of LCCBM hydrates. The four simulated models were named A0, A1, A2, and A3, and the initial structural models are shown in Figure 10. To facilitate observation, the hydrogen atoms in the promoter molecules and CH_4_ molecules in the model are hidden. Table 1 lists the information required for all the different models constructed. (COD 3000410.cif, 3000411.cif, and 3000412.cif contain the supplementary crystallographic data for this paper. These data can be obtained free of charge until 7 February 2023 via http://www.crystallography.net/cod/search.html).

### 3.2. Type of Force Field

The force field is the core of the simulation operation. The CVFF force fields are usually used for small organic (amide, carboxylic acid, etc.) crystals and gas phase structures. It can handle peptides, proteins, and various organic compounds. It has been widely used for many years, especially for structural and binding energy studies. Liu et al. [38] and Wang et al. [39] calculated the intermolecular forces for all molecules in hydrates using the CVFF force field. In the CVFF, the water model for the potential function is the simple point charge (SPC) when the main molecule of the cage frame of the hydrate is considered to be a rigid molecule, i.e., with a fixed bond length and bond angle. The single point charges on the O and H atoms of the H_2_O molecule are +0.41 e and −0.82 e, respectively; the C and H atoms of the CH_4_ molecule have charges of −0.4 e and +0.1 e, respectively. The energy expression for CVFF is given below [40,41,42]:

(4) $$\begin{array}{c} E=\sum_{b}D_{b}{\left[1-e^{-\partial \left(b-b_{0}\right)}\right]}^{2}+\sum_{\theta }H_{\theta }{\left(\theta -\theta_{0}\right)}^{2}+\sum_{\varphi }H_{\varphi }\left[1-\mathrm{scos}\left(n\varphi \right)\right]+\\ \sum_{X}H_{X}X^{2}+\sum_{b}\sum_{b^{\prime }}F_{bb^{\prime }}\left(b-b_{0}\right)\left(b^{\prime }-b_{0}^{\prime }\right)\\ +\sum_{\theta }\sum_{\theta^{\prime }}F_{\theta \theta^{\prime }}\left(\theta -\theta_{0}\right)\left(\theta^{\prime }-\theta_{0}^{\prime }\right)+\\ \sum_{b}\sum_{\theta }F_{b\theta }\left(b-b_{0}\right)\left(\theta -\theta_{0}\right)+\sum_{\varphi }F_{\varphi \theta \theta^{\prime }}\cos\varphi \left(\theta -\theta_{0}\right)\left(\theta^{\prime }-\theta_{0}^{\prime }\right)+\\ \sum_{X}\sum_{X^{\prime }}F_{XX^{\prime }}XX^{\prime }+\sum \varepsilon \left[{\left(\frac{r^{*}}{r}\right)}^{12}-2{\left(\frac{r^{*}}{r}\right)}^{6}\right]+\frac{q_{i}q_{j}}{\varepsilon r_{ij}} \end{array}$$

where *D*, *H*, and *F* stand for the force constants, and *b*, *θ*, and *X* denote the bond length, bond angle, out-of-plane parameter, and dihedral angle, respectively. The zero condition represents the equilibrium value of that parameter. *r_ij_* refers to the distance between particle *i* with charge *q_i_* from particle *j* with charge *q_j_*, *ε* introduces the well depth in van der Waals interaction term, and s and n are the sign convention and nonnegative integer coefficient parameters for the dihedral term, respectively.

In the formula, Terms 1–4 represent the energy of deformation of bond lengths, bond angles, torsion angles, and out-of-plane interactions, respectively. Terms 5–9 represent couplings between deformations of internal coordinates. Terms 10–11 describe the non-bond interactions.

### 3.3. Simulation Scheme

The CVFF force field was employed to explain the molecular interactions in the LCCBM hydrate clathrate. To obtain a stable structure, a steepest-descent algorithm and the conjugate gradient algorithm [42] were used to minimize the energy of the initial model system. The constant volume and constant temperature (NVT) ensemble was performed for 100 ps to reach each targeted temperature. The constant pressure and constant temperature (NPT) ensemble MD simulations were carried out to demonstrate the hydrates’ stability and a total simulation time of 500 ps (energy and temperature stabilization around 500 ps, in which the system reaches equilibrium). The simulated pressure was set to P = 2 MPa, and the temperature was set to T = 273 K, 283 K, and 293 K, respectively. In addition, we also simulated the potential energy change of the A3 model at a higher decomposition temperature T = 303 K. The van der Waals and long-range Coulomb interaction terms were calculated using the Ewald summation method [43]. The Nose–Hoover [44,45] and Berendsen methods were used to control the system temperature and pressure. The equations of motion were solved by the Verlet leap-frog algorithm [46] with a time step set to 1 fs. The van der Waals interaction calculation method was based on atoms, the cutoff radius was set to 1.5 nm, and the summation accuracy was set to 10^−6^ kJ/mol.

## 4. Conclusions

In this research work, we systematically investigated the effect of three cyclic promoters on the stability of LCCBM hydrates using the Materials Studio software (v. 2020), and molecular dynamics (MD) simulations were performed using the consistent valence force field (CVFF). The main findings can be summarized as follows:

(1) At P = 2 MPa and T = 273 K, the stability of the LCCBM hydrate in the pure water system without any promoter was poor, while CP, CP-one, and CP-ol all showed good promotion of LCCBM hydrate with 30% CH_4_ cage occupancy;

(2) The LCCBM hydrate containing CP maintains good stability when the pressure remains constant and the temperature increases to T = 293 K. The hydrate model containing CP-one maintains the basic stability of the hydrate structure, although the system disorder increases significantly with increasing temperature. Meanwhile, the hydrate model containing CP-ol destroys the cage structure of the hydrate at T = 293 K due to the formation of new hydrogen bonds between the water molecules in the cage and the hydrophilic hydroxyl groups of the CP-ol molecules, and the encapsulated CH_4_ and N_2_ guest molecules escape from the destroyed water cage and are distributed in bubbles in aqueous solution;

(3) The decomposition time of the LCCBM hydrate containing CP-ol ranges from 100 to 300 ps at P = 2 MPa and T = 293 K, which changes to 25–95 ps when the temperature rises to 303 K. This indicates that the higher the decomposition temperature of the hydrate, the earlier the decomposition starts and the shorter the time required for complete decomposition.

## Figures and Tables

**Table 1:** The LCCBM hydrate model.

SII LCCBM Hydrate Model	5^12^	5^12^6^4^	CH_4_ Molecule Occupancy (%)
A0	38 CH_4_ 90 N_2_	0	30
A1	38 CH_4_ 90 N_2_	64 CP	30
A2	38 CH_4_ 90 N_2_	64 CP-one	30
A3	38 CH_4_ 90 N_2_	64 CP-ol	30

## Data Availability

Not applicable.

## Sample Availability

Samples of the compounds LCCBM hydrate models are available from the authors.
